# Pragmatic randomised trial of a 12-week exercise and nutrition program for Aboriginal and Torres Strait Islander women: clinical results immediate post and 3 months follow-up

**DOI:** 10.1186/1471-2458-12-933

**Published:** 2012-10-31

**Authors:** Karla Canuto, Margaret Cargo, Ming Li, Katina D’Onise, Adrian Esterman, Robyn McDermott

**Affiliations:** 1 Sansom Institute for Health Research, University of South Australia, Adelaide, Australia

**Keywords:** Aboriginal, Torres strait islander, Physical activity, Women, Lifestyle program, Health promotion

## Abstract

**Background:**

Aboriginal and Torres Strait Islander women experience higher rates of heart disease and type 2 diabetes than non-Indigenous Australian women. Increasing physical activity, improving diets and losing weight have been shown to reduce cardio metabolic risk. The primary aim was to evaluate the effectiveness of a 12-week structured exercise and nutrition program in a cohort of urban Indigenous Australian women on waist circumference, weight and biomedical markers of metabolic functioning from baseline (T1) to program completion (T2). The secondary aim assessed whether these outcomes were maintained at 3-month follow-up.

**Methods:**

One hundred Aboriginal and/or Torres Strait Islander women aged 18–64 years living in the Adelaide metropolitan area were recruited. The program included two 60-minute group cardiovascular and resistance training classes per week, and four nutrition education workshops. Participants were randomly assigned to an ‘active’ group or ‘waitlisted’ control group. Body weight, height, waist and hip circumference, blood pressure, fasting glucose, fasting insulin, glycated haemoglobin (HbA1C), lipid profile and C-reactive protein (CRP) were assessed at baseline (T1), immediately after the program (T2) and three months post program (T3).

**Results:**

The active group showed modest reductions in weight and body mass index (BMI). Compared to the waitlisted group, the active group had a statistically significantly change in weight and BMI from baseline assessments; at T2, ^-^1.65 kg and ^-^0.66 kg/m^2^ and at T3, ^-^2.50 kg and ^-^1.03 kg/m^2^, respectively. Systolic and diastolic blood pressure also had a statistically significant difference from baseline in the active group compared to the waitlisted group at T2, ^-^1.24 mmHg and ^-^2.46 mmHg and at T3, ^-^4.09 mmHg and ^-^2.17 mmHg, respectively. The findings were independent of the baseline measure of the outcome variable, age, households with children and employment status. Changes in waist circumference and other clinical measures were not significant at T2 or T3. The primary outcome measure, waist circumference, proved problematic to assess reliably. Missing data and participants lost to follow-up were significant.

**Conclusions:**

This 12-week exercise program demonstrated modest reductions in weight, BMI and blood pressure at T2, which improved further at 3-month follow-up (T3). Positive intervention effects were observed despite low attendance at exercise classes. Structured exercise programs implemented in community settings require attention to understanding the barriers to participation for this high risk group.

**Trial registration:**

Australian New Zealand Clinical Trials Registry ACTRN12610000224022

## Background

The Australian National Aboriginal and Torres Strait Islander Health Survey (NATSIHS) and the National Health Survey (NHS) were conducted between 2004 and 2005. The data were analysed and reported in the Aboriginal and Torres Strait Islander Health Performance Framework 2010 [1]. The rates of obesity and type 2 diabetes mellitus (T2DM) reported for Australian Indigenous women were much higher compared to non-Indigenous women. Rates of obesity based on body mass index (BMI) derived from self-reported height and weight were 36.8% for Indigenous women, and significantly higher than non-Indigenous women (16.7%) [1]. Self-reported T2DM was 11% for Indigenous women compared to 3% of non-Indigenous women in non-remote areas [1]. The NATSIHS & NHS surveys also reported that 53% of Indigenous women are sedentary, which is significantly more than the 34% of non-Indigenous women [1]. Young Indigenous women, aged 15–34 years, significantly increased their weight and waist circumference during a 7-year longitudinal study in Far North Queensland. Aboriginal women gained an average of 1.5 kg (kg) in weight and 1.6 cm (cm) around their waist per year while the Torres Strait Islander women gained an average of 1.2 kg and increased their waist by 1.2 cm each year [2]. The life expectancy gap between Indigenous and non-Indigenous women is currently estimated at ten years [3].

Research consistently supports that regular physical activity is effective in the primary and secondary prevention of many chronic diseases, including diabetes [4]. Programs incorporating physical activity and nutrition education have been shown to reduce body weight [5], blood pressure [6,7] the incidence of T2DM in 'at risk' participants [8] and improve glycemic control and reduce diabetes-related complications in diagnosed patients [9,10].

Implementing and evaluating well designed physical activity and nutrition education studies targeting Indigenous populations is difficult and consequently the evidence limited [9,11]. A 4-month structured physical activity program for Maori men and women reported a decrease in weight and body mass index (BMI) and an improvement in systolic and diastolic blood pressure [12]. However, the study did not include a control group and consisted of only 30 participants. The Community Health Assessment and Promotion Project (CHAPP) was a 10-week nutrition and physical activity program with 72 ‘black’ female participants aged 18 – 59 [13]. CHAPP also reported favourable results for weight loss and blood pressure but utilised a pre-post design with no control group [13].

The Aboriginal and Torres Strait Islander Women’s fitness program was informed by a pilot project, the ‘Thursday Island Women’s Fitness Challenge’, which was undertaken in a remote setting. The pilot study was an 8-week program that included structured group circuit and aqua aerobics classes conducted twice a week. The study did not include a control group. Participants attending the post program assessment (n=10) lost 4.93 cm from their waist and 0.03 kg in weight. Blood pressure was not assessed [14].

This study aimed to implement and evaluate the effectiveness of the 12-week structured exercise and nutrition education program in a cohort of urban Indigenous Australian women on waist circumference, weight and biomedical markers of metabolic functioning. We hypothesised that the active group who participated in the intervention would achieve a significant reduction in waist circumference and improved metabolic functioning compared to the waitlisted group immediately following the intervention and three months following program completion.

## Methods

### Participants

Aboriginal and Torres Strait Islander women from the Adelaide metropolitan area were invited to participate in the program. Women were eligible if they identified as being of Aboriginal and/or Torres Strait Islander descent, aged between 18 and 64 years, and had a waist circumference greater than 80 cm. Participants were excluded if they were pregnant or were deemed physically unable to participate by their doctor. The recruitment process is described in detail elsewhere [15].

**Figure 1:**
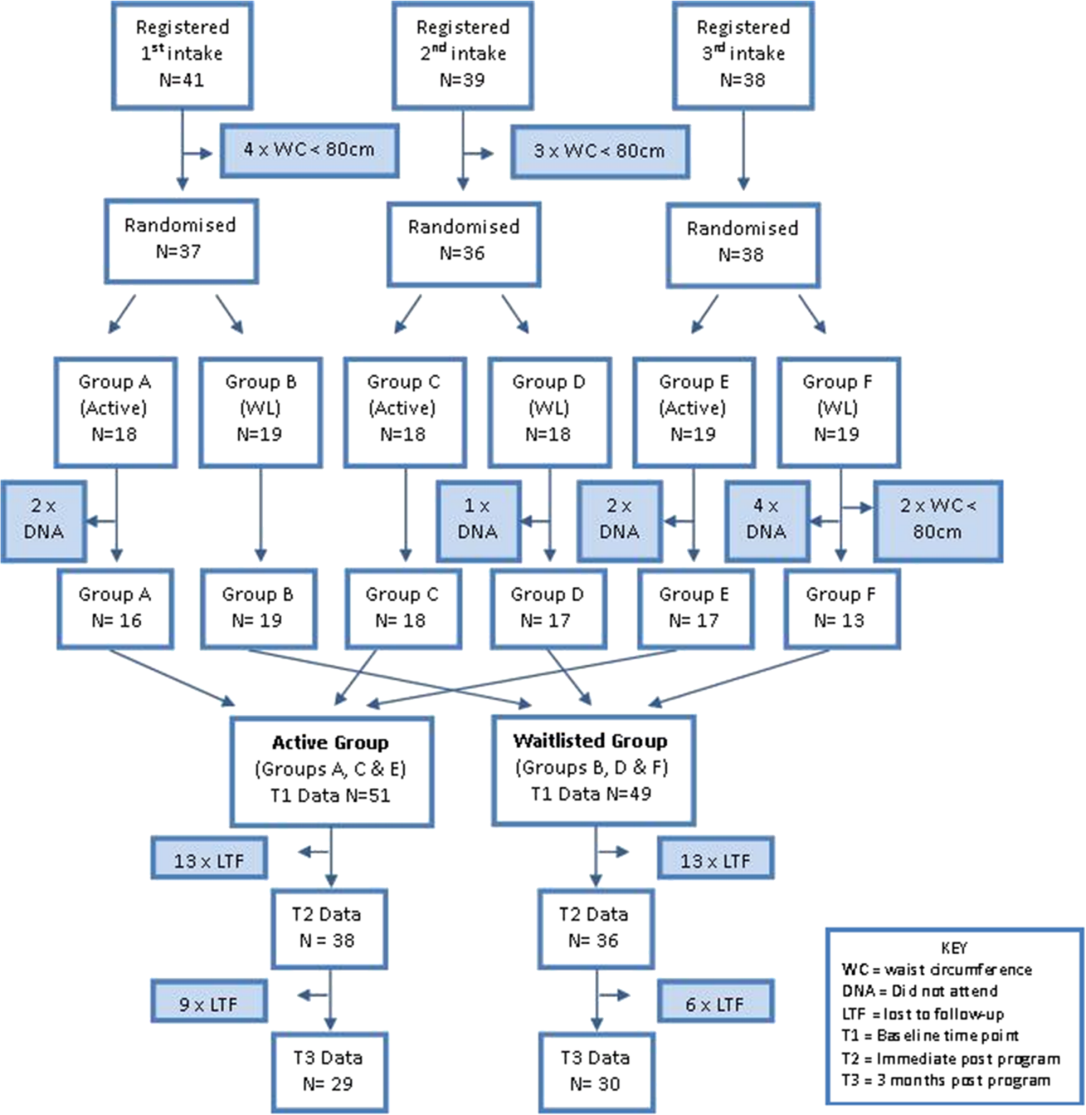
**Participant flow chart.** Participant numbers from registration through to the allocation of groups and their participation in the three assessments time points (T1, T2 and T3).

Participants were recruited in three intakes over approximately 13 months starting in January 2010. Figure 1 shows the pathway of participants from registration to group assignment and baseline assessment. A total of 119 women registered to participate in the program. One participant was ineligible due to pregnancy however she re-registered for the second intake after the birth of her child. Each intake was randomised to form two groups of even size; an ‘active’ and ‘waitlisted’ group. Each group was assigned a letter, with A, C and E being the ‘active’ groups and B, D and F being the ‘waitlisted’ groups. Attempts were made to assess participant’s baseline waist circumference before randomly assigning them to the ‘active’ or ‘waitlisted’ groups. Nine participants did not meet the waist circumference eligibility criteria, seven of whom were excluded before randomisation and an additional two participants who had been assigned to group F. Nine participants failed to attend their baseline anthropometric assessment and were excluded from analysis.

Some participants were lost to follow-up (LTF) and/or chose not to participate in all assessments at all assessment time points. Participants were considered ‘lost to follow-up’ if they did not attend any part of the T3 assessments or subsequent assessments. The results reported here include the assessment at baseline (T1), immediately post program (T2) and 3 months post program (T3). These three assessments are approximately three months apart.

All eligible participants attended the baseline anthropometric assessment however fasting blood tests were not all completed and missing data increased over subsequent assessment time points. The completion rates of the anthropometric and pathology assessments are summarised in Table 1.

**Table 1:** Number and percentage of participants who attended anthropometric and pathology assessments at T1, T2, and T3

	**Anthropometrics N (%)**		**Pathology N (%)**	
	**Waitlisted**	**Active**	**Waitlisted**	**Active**
T1	49 (100)	51 (100)	29 (59)	44 (86)
T2	35 (71)	36 (71)	26 (53)	23 (45)
T3	29 (59)	27 (53)	23 (47)	49 (49)

Percentages are calculated on the total number of participants at T1.

### Program

The Aboriginal and Torres Strait Islander Women’s Fitness Program is a structured 12-week group program. There are four key components of the intervention; the group exercise classes, incidental activity and walking, nutrition workshops and positive reinforcement and encouragement. During the 12-week intervention participants were encouraged to attend two group exercise sessions per week facilitated by qualified female fitness instructors. The exercise sessions were implemented as outlined in the facilitator manual. Each exercise session incorporated both aerobic and resistance training for 60 minutes. Participants were also encouraged to aim for 10,000 steps per day. Steps were assessed using Yamax SW200 pedometers (Yamax Corp., Tokyo) and were self-recorded in an exercise diary provided. During the 12-week program four 1-hour nutrition workshops were delivered by a female dietician. Nutrition topics included food label reading, recipe modification and a cooking demonstration. Fortnightly newsletters were distributed to participants. Each newsletter included a healthy recipe, nutrition and exercise tips and acknowledged the participants from their group logging the most steps since the previous newsletter.

Waitlisted women received the same pedometer and exercise diary as the active group. To keep the waitlisted group engaged, they also received monthly newsletters with a recipe and tips on physical activity and healthy eating and were invited to attend four 1-hour workshops facilitated by a dietician. Waitlisted participants were offered the structured physical activity program approximately 12 months after their baseline assessment. The program is described in more detail elsewhere [15].

### Outcome measures

Participant’s demographic data was collected via a questionnaire completed during the baseline assessment period (T1). Participants reported employment status, household income and highest educational attainment. Some categories were collapsed due to low numbers. The number of children and number of adults in the household were separate, open ended questions. For regression analysis dichotomous categories for ‘kids in the household’ (no children and at least one child) and employed (unemployed and employed) were created. Participants were asked to identify their ethnicity (Aboriginal and/or Torres Strait Islander). All participants identified as Aboriginal except for two women who identified as being both of Aboriginal and Torres Strait Islander descent. Analyses were not adjusted for ethnicity.

Height, weight, waist and hip circumference, blood pressure and fasting venous blood samples were collected from participants at baseline (T1), immediately after the program (T2) and three months after the completion of the program (T3). The average time period between T1 and T2 was 112 days (95% confidence interval (CI) 109–115 days). The average time period between T1 and T3 was 189 days (95% CI, 187–192).

Anthropometric assessments were conducted using the protocols developed by the International Society for the Advancement of Kinanthropometry (ISAK) [[16]]. An ISAK accredited assessor measured participant’s height, weight, waist and hip circumference which were all measured twice and were averaged. If the two measurements were not within the acceptable error range (3%), a third assessment was taken and the outlier dismissed. Blood pressure was taken three times at each assessment using a digital Ormon T8 (JA Davey Pty Ltd; Melbourne, Vic, Australia). An average systolic and diastolic blood pressure was calculated using all available measurements. These assessments are explained in detail in the study protocol [15].

The pathology company, Healthscope Inc., collected and analysed the fasting blood samples. Participants could get their blood pathology test by attending one of the 51 Adelaide pathology clinics in their own time, by home visit or during the anthropometric assessment when a phlebotomist was present. Fasting blood glucose was analysed using the Hexokinase assay with Siemens Advia 2400 (Siemens Healthcare Diagnostics Inc., New York, USA). Fasting serum insulin, total cholesterol, high-density lipoprotein (HDL) and triglyceride concentrations were analysed using Chemiluminescent Immunoassay with Siemens Advia 2400. Glycated haemoglobin (HbA1C) was analysed using the Immunoturbidimetry assay with Roche Integra 800 (Roche Diagnostics Ltd. West Sussex, UK). C-reactive protein (CRP) was analysed using the Immunoturbidimetry assay with Siemens Advia 2400. For analysis, CRP values greater than 100 mg/L were replaced with missing values as this indicates a bacterial infection rather than a chronic underlying inflammatory state. Low-density lipoprotein (LDL) was calculated by Healthscope Inc. Homeostatic model assessment for insulin resistance (HOMA-IR) is calculated using the formula:

HOMA-IR = (fasting glucose [mmol/L] × fasting insulin [μU/mL])/22.5.

Diabetes status was determined by self-reported diagnosis of type 2 diabetes or if participants reported taking T2DM medications. Additionally participants were classified as having T2DM if their fasting glucose was greater than 7.0 mmol/L at their baseline pathology assessment, in accordance with the World Health Organization diagnostic criteria [17].

Participation in the exercise classes and nutrition workshops was recorded by project staff. Fidelity was assessed at each exercise class by a project staff member (not by the fitness instructor). The fidelity tool assessed the completion of and any modifications of the program for each major component of the class.

### Ethics

This project received ethics approval from the Human Research Ethic Committees of the University of South Australia (reference number P006/09) and the Aboriginal Health Council of South Australia (reference number 04-09-298).

### Community consultation

The project has been given advice and guidance from their collaborating partners, Nunkuwarrin Yunti of South Australia Inc and the Aboriginal Sobriety Group Inc. in Adelaide, and a specially formed advisory committee of local Aboriginal and Torres Strait Islander women.

### Statistical analysis

Data analysis was performed in Stata11 [18]. The demographic characteristics are summarised in Table 2 and clinical outcomes, including diabetes status, displayed in Table 3. These tables illustrate the similarities and differences between the two randomly assigned groups. The descriptive analyses of demographics and baseline assessments were repeated by group and follow-up status in Tables 4 and 5 to explore the differences between participants lost to follow and those with T3 data for both groups. The effect of program participation on all anthropometric, blood pressure and pathology results was conducted using linear regression (Tables 6 and 7). Linear regression was performed on the change of each variable between time points, firstly from baseline (T1) to immediately post program (T2) (Table 6), then between baseline (T1) and 3-month post program (T3) (Table 7). This was done with and without adjusting for potential confounders. Tables 6 and 7 show regression analysis with four models; model 1 is crude without adjustments, model 2 adjusts for age and baseline measure of the outcome variable, model 3 adjustments for age and baseline measure of the outcome variable and demographic confounders, and model 4 builds on model 3 with T2DM status included. The demographic confounders are employment status and kids in the household which were both collapsed into dichotomous variables and any missing data were dropped. These demographic confounders were chosen because of the uneven distribution of the characteristic by group at baseline. The sample size calculation is reported in the protocol paper [15].

**Table 2:** Demographics at baseline by group

		**Waitlisted group N=49**		**Active group N=51**	
		**No. (%)**	**95% CI**	**No. (%)**	**95% CI**
Employment	unemployed	9 (18)	7.5-30.0	16 (31)	18.8-45.2
	Part time	11 (22)	10.7-35.1	9 (18)	7.1-28.9
	Full time	28 (57)	44.1-72.6	25 (49)	35.8-64.2
	Unanswered	1 (2)	—	1 (2)	—
Education (highest attained)	< year 12	13 (35)	19.3-51.0	11 (25)	11.9-38.1
	Year 12	4 (11)	0.5-21.1	7 (16)	4.8-27.0
	TAFE	8 (16)	8.0-35.3	17 (33)	23.9-53.4
	University	12 (24)	16.9-48.0	9 (18)	8.2-32.7
	Unanswered	12 (24)	—	7 (14)	—
Gross household income	<$40,000	14 (29)	21.1-52.9	15 (29)	19.2-47.5
	$40,000-$60,000	13 (27)	18.7-49.7	13 (25)	15.3-42.5
	$60,000	11 (22)	14.1-43.8	17 (33)	23.2-52.3
	Unanswered	11 (22)	—	6 (12)	—
No. of adults in the household	1	15 (31)	23.5-55.5	13 (25)	16.1-44.3
	2	9 (18)	9.8-37.6	20 (39)	31.2-61.8
	3	9 (18)	9.8-37.6	4 (8)	0.4-18.2
	≥4	5 (10)	2.1-24.2	6 (12)	3.3-24.6
	Unanswered	11 (22)	—	8 (16)	—
No. of children in the household	0	11 (22)	14.1-43.8	23 (45)	38.2-68.8
	1	9 (18)	7.7-34.4	5 (10)	1.8-21.5
	2	9 (18)	5.7-31.1	6 (12)	3.3-24.6
	3	7 (14)	7.7-34.4	6 (12)	3.3-24.6
	≥4	4 (8)	0.5-20.6	3 (6)	−0.8-14.8
	Unanswered	9 (18)	—	8 (16)	—

Questions that a participant left blank or the response could not be interpreted have been coded as “unanswered”.

Abbreviations: TAFE = “Technical And Further Education” or vocational studies.

**Table 3:** Baseline mean anthropometric and blood measurements by group

		**Waitlisted N=49**		**Active N=51**	
		**Mean**	**95% CI**	**Mean**	**95% CI**
Age	Age (years)	40.7	37.7-43.6	39.8	36.7-43.1
Anthropometrics	Height (m)	1.62	1.61-1.64	1.61	1.60-1.62
	Weight (kg)	88.8	82.8-94.8	94.0	88.1-99.9
	BMI (kg/m^2^)	33.5	31.5-35.6	36.1	34.0-38.2
	Waist (cm)	101.5	97.0-106.0	104.2	100.0-108.3
	Hip (cm)	117.0	112.3-121.7	120.6	115.9-125.2
	Waist to Hip Ratio	0.87	0.85-0.89	0.87	0.85-0.88
Blood Pressure	Systolic BP (mmHg)	129.5	124.1-135.0	129.4	125.4-133.4
	Diastolic BP (mmHg)	87.5	83.9-91.1	88.3	85.3-91.3
Pathology	Glucose (mmol/L)	6.4	5.1-7.7	5.6	4.9-6.3
	Insulin (mU/L)	17.4	14.1-20.7	17.5	14.7-20.2
	HbA1c (%)	6.5	6.0-7.1	6.1	5.7-6.4
	Cholesterol (mmol/L)	4.6	4.3-5.0	4.9	4.6-5.2
	Triglycerides (mmol/L)	1.5	1.1-1.8	1.5	1.3-1.7
	LDL (mmol/L)	2.8	2.4-3.0	3.0	2.8-3.2
	HDL (mmol/L)	1.2	1.1-1.3	1.2	1.1-1.3
	LDL:HDL	2.4*	2.1-2.7	2.6	2.3-2.9
	Cholesterol:HDL	4.1	3.6-4.5	4.2	3.8-4.5
	CRP (mg/L)	7.2	4.4-10.0	7.5^#^	5.6-9.4
Calculated HOMA-IR	HOMA-IR	5.3	3.4-7.2	4.3	3.6-5.1

The observations for blood pressure were reduced due to missing data; waitlisted participants n=45, active participants n=49. The observations for pathology results were reduced due to missing data; waitlisted participants n=29, active participants n=44.

* Observation reduced by one. LDL value missing – invalid as triglyceride concentration was > 4.5 mmol/L.

# Observation reduced by one. CRP was > 100 mg/L indicating bacterial infection.

Abbreviations: CI = confidence intervals; BMI = body mass index; Waist = waist circumference; Hip = hip circumference; HbA1C = glycated haemoglobin; LDL = low-density lipoprotein; HDL = high-density lipoprotein; CRP = C-reactive protein; HOMA-IR = Homeostatic model assessment for insulin resistance.

**Table 4:** Participant demographics and T2DM status at baseline by group and T3 follow-up status

		**Waitlisted group N=49**		**Active group N=51**	
		**Followed-up N=30% (95% CI)**	**LTF at T3 N=19% (95% CI)**	**Followed-up N=29% (95% CI)**	**LTF at T3 N=22% (95% CI)**
Employment	unemployed	20.0 (5–35)	15.8 (0–34)	17.2 (3–32)	50.0 (27–73)
	Part time	26.7 (10–43)	15.8 (0–34)	20.7 (5–36)	13.6 (0–29)
	Full time	53.3 (34–72)	63.2 (39–87)	58.6 (40–78)	36.4 (15–58)
	Unanswered	—	5.3 (0–16)	3.4 (0–11)	—
Education	< year 12	20.0 (5–35)	36.8 (13–61)	24.1 (8–41)	18.2 (1–36)
	Year 12	10.0 (0–21)	5.3 (0–16)	10.3 (0–22)	18.2 (1–36)
	TAFE	26.6 (10–43)	—	27.6 (10–45)	40.9 (19–63)
	University	23.3 (7–39)	26.3 (5–48)	24.1 (8–41)	9.1 (0–2)
	Unanswered	20.0 (5–35)	31.6 (9–55)	13.8 (0–27)	13.6 (0–29)
Income	<$40,000	16.0 (1–31)	35.7 (7–64)	8.3 (0–20)	12.5 (0–31)
	$40,000-$60,000	32.0 (12–52)	28.6 (2–56)	29.2 (10–49)	37.5 (11–64)
	$60,000	28.0 (9–47)	—	50.0 (28–72)	31.2 (6–57)
	Unanswered	24.0 (6–42)	35.7 (7–64)	12.5 (0–27)	19.0 (0–40)
No. of adults in the household	1	30.0 (13–47)	32 (9–55)	27.6 (10–45)	22.7 (4–42)
	2	16.7 (3–31)	21.1 (9–41)	41.4 (22–60)	36.4 (15–58)
	3	26.7 (10–43)	5.3 (0–16)	13.8 (0–27)	18.0 (1–36)
	≥4	6.7 (0–16)	16.0 (0–34)	6.9 (0–17)	—
	Unanswered	20.0 (5–35)	26.3 (5–48)	10.3 (0–22)	22.7 (4–42)
No. of children in the household	0	20.0 (5–35)	26.3 (5–48)	37.9 (19–57)	54.5 (32–77)
	1	16.7 (3–31)	21.0 (1–41)	17.2 (3–32)	—
	2	20.0 (5–35)	15.8 (0–34)	17.2 (3–32)	4.5 (0–14)
	3	16.7 (3–31)	10.5 (0–26)	13.8 (0–27)	9.1 (0–22)
	≥4	6.7 (0–16)	10.5 (0–26)	3.4 (0–11)	9.1 (0–22)
	Unanswered	20.0 (5–35)	15.8 (0–34)	10.3 (0–22)	22.7 (4–42)
Diabetes*	Diabetic	26.7 (10–43)	21.1 (2–40)	17.2 (3–32)	13.6 (1–29)
	non-diabetes	73.3 (57–89)	78.9 (60–98)	82.8 (68–97)	86.4 (71–101)

Questions that a participant left blank or the response could not be interpreted have been coded as “unanswered”.* Diabetes was defined by self-reported diagnosis, self-reported prescription of T2DM medications or fasting glucose > 7.0 mmol/L at T1, in accordance with the World Health Organization diagnostic criteria [17].

Abbreviations: TAFE = “Technical And Further Education” or vocational studies; LTF = lost to follow-up.

**Table 5:** Baseline age and clinical markers by group and T3 follow-up status

	**Waitlisted group N=49**		**Active group N=51**	
	**Followed-up N=30 Mean (95% CI)**	**LTF at T3 N=19 Mean (95% CI)**	**Followed-up N=29 Mean (95% CI)**	**LTF at T3 N=22 Mean (95% CI)**
Age (year)	41.7 (38.1-45.3)	39.1 (33.6-44.5)	43.5 (39.6-47.4)	35.1 (30.2-40.1)
Weight (kg)	94.8 (86.3-103.4)	79.2 (73.3-85.0)	92.6 (84.5-100.6)	95.9 (86.5-105.2)
BMI (kg/m^2^)	35.5 (32.5-38.5)	30.4 (28.4-32.5)	35.5 (32.5-38.5)	36.8 (33.8-39.9)
WC (cm)	106.4 (100.0-112.8)	93.8 (89.6-98.0)	103.2 (97.9-108.6)	105.4 (98.5-112.4)
Hip (cm)	121.7 (115.1-128.4)	109.4 (104.5-114.3)	120.0 (113.3-126.7)	121.3 (114.6-128.1)
Systolic BP (mmHg)	129.5 (121.9-137.0)	129.7 (121.3-138.1)	129.1 (123.9-134.3)	129.9 (123.0-136.8)
Diastolic BP (mmHg)	88.1 (83.2-92.9)	86.6 (80.7-82.4)	87.2 (83.6-90.8)	89.9 (84.3-95.4)
Glucose (mmol/L)	6.6 (5.0-8.2)	5.6 (3.5-7.7)	5.8 (4.7-6.8)	5.2 (4.5-5.9)
Insulin (mU/L)	16.1 (12.9-19.3)	22.3 (10.1-34.5)	17.2 (13.7-20.7)	18.6* (13.2-24.1)
HbA1c (%)	6.6 (5.9-7.2)	6.4 (5.5-7.3)	6.2 (5.7-6.6)	6.0* (5.4-6.6)
Cholesterol (mmol/L)	4.6 (4.3-5.0)	4.6 (3.7-5.4)	5.2 (4.9-5.6)	4.2 (3.9-4.6)
Triglycerides (mmol/L)	1.6 (1.1-2.0)	1.1 (0.8-1.5)	1.7 (1.4-2.0)	1.2 (0.8-1.5)
LDL (mmol/L)	2.8* (2.4-3.1)	2.7 (1.8-3.6)	3.3 (3.0-3.6)	2.5 (2.2-2.8)
HDL (mmol/L)	1.1 (1.0-1.2)	1.3 (1.0-1.6)	1.2 (1.1-1.3)	1.2 (1.0-1.3)
CRP (mg/L)	7.6 (4.2-11.0)	5.6 (1.1-10.2)	6.2 (3.8-8.6)	10.2^#^ (7.5-12.9)

The observations for blood pressure were reduced due to missing data; waitlisted participants followed up n=27, LTF n=18, active participants LTF n=20. The observations for pathology results were reduced due to missing data; waitlisted participants followed up n=23, LTF n=6, active participants LTF n=15.

* Observation reduced by one. LDL value missing – invalid as triglyceride concentration was > 4.5 mmol/L.

# Observation reduced by one. CRP was > 100 mg/L indicating bacterial infection.

Abbreviations: CI = confidence intervals; BMI = body mass index; Waist = waist circumference; Hip = hip circumference; HbA1C = glycated haemoglobin; LDL = low-density lipoprotein; HDL = high-density lipoprotein; CRP = C-reactive protein.

**Table 6:** Regression of mean change in outcome variables from baseline to immediate post program (T1-T2)

	**N**	**Model 1**		**p value**	**Model 2**		**p value**	**Model 3**		**p value**	**Model 4**		**p value**
		**β coeff**	**95% CI**		**β coeff**	**95% CI**		**β coeff**	**95% CI**		**β coeff**	**95% CI**	
Weight (kg)	71	−1.85	−3.43- -0.27	0.022	−1.84	−3.44- -0.24	0.025	−1.56	−3.15-0.04	0.055	−1.65	−3.27- -0.03	0.046
BMI (kg/m^2^)	71	−0.76	−1.35- -0.16	0.013	−0.76	−1.36- -0.16	0.015	−0.64	−1.24- -0.04	0.036	−0.66	−1.27- -0.05	0.035
Waist (cm)	71	−1.36	−3.26-0.54	0.158	−1.45	−3.37-0.46	0.134	−1.39	−3.36-0.59	0.166	−1.50	−3.50-0.51	0.140
Hip (cm)	71	−0.71	−2.52-1.09	0.434	−0.79	−2.53-0.95	0.371	−0.83	−2.60-0.94	0.353	−0.84	−2.65-0.97	0.357
Systolic BP (mmHg)	66	−0.19	−6.55-6.17	0.952	−0.99	−6.57-4.58	0.723	−1.75	−7.53-4.02	0.546	−1.24	−7.09-4.62	0.674
Diastolic BP (mmHg)	66	−1.64	−6.71-3.42	0.519	−1.95	−6.33-2.43	0.377	−2.15	−6.69-2.38	0.347	−2.46	−7.09-2.17	0.293
Insulin (mU/L)	44	−1.91	−7.18-3.36	0.468	−1.65	−7.07-3.76	0.540	−1.26	−6.72-4.20	0.643	0.48	−4.80-5.77	0.854
Glucose (mmol/L)	44	0.33	−1.03-1.69	0.628	−0.54	−1.57-0.50	0.300	−0.64	−1.67-0.39	0.217	−0.47	−1.42-0.47	0.316
HbA1c (%)	44	−0.14	−0.37-0.09	0.221	−0.04	−0.25-0.18	0.723	−0.05	−0.27-0.17	0.646	−0.04	−0.25-0.18	0.741
Cholesterol (mmol/L)	44	−0.10	−0.48-0.29	0.619	0.03	−0.36-0.42	0.877	0.032	−0.38-0.44	0.878	0.00	−0.42-0.42	0.994
Triglycerides (mmol/L)	44	−0.03	−0.30-0.24	0.847	−0.03	−0.28-0.22	0.787	−0.03	−0.29-0.23	0.812	0.02	−0.24-0.28	0.879
LDL (mmol/L)	43	0.00	−0.31-0.31	0.998	0.08	−0.24-0.39	0.617	−0.03	−0.29-0.23	0.812	0.05	−0.27-0.37	0.755
HDL (mmol/L)	44	−0.06	−0.15-0.03	0.194	−0.03	−0.11-0.05	0.453	−0.03	−0.11-0.06	0.511	−0.04	−0.12-0.05	0.410
CRP (mg/L)	44	−0.80	−3.73-2.14	0.587	−0.55	−3.48-2.38	0.705	−0.69	−3.62-2.23	0.635	−0.57	−3.63-2.50	0.710

Model 1 is crude and unadjusted. Model 2 is adjusted for age at baseline and the baseline assessment of the respective outcome measure. Model 3 is adjusted for the confounders in Model 2 plus kids in the household and employment status. Model 4 adjusts for all potential confounders in model 3 plus T2DM status.

Abbreviations: CI = confidence intervals; BMI = body mass index; Waist = waist circumference; Hip = hip circumference; HbA1C = glycated haemoglobin; LDL = low-density lipoprotein; HDL = high-density lipoprotein; CRP = C-reactive protein.

**Table 7:** Regression of mean change in outcome variables from baseline to 3 months post program (T1-T3)

	**N**	**Model 1**		**p value**	**Model 2**		**p value**	**Model 3**		**p value**	**Model 4**		**p value**
		**β coeff**	**95% CI**		**β coeff**	**95% CI**		**β coeff**	**95% CI**		**β coeff**	**95% CI**	
Weight (kg)	56	−2.17	−4.09- -0.25	0.028	−2.13	−4.11- -0.15	0.036	−2.20	−4.16-0.24	0.029	−2.50	−4.46- -0.54	0.013
BMI (kg/m^2^)	56	−0.95	−1.69- -0.21	0.013	−0.92	−1.68- -0.16	0.019	−0.91	−1.68- -0.15	0.020	−1.03	−1.79- -0.27	0.009
Waist (cm)	56	−1.81	−4.31-0.69	0.153	−1.60	−4.19-0.99	0.220	−1.49	−4.12-1.14	0.261	−1.50	−4.20-1.19	0.268
Hip (cm)	56	−1.16	−3.73-1.41	0.370	−1.44	−4.00-1.13	0.265	−1.52	−3.98-0.95	0.223	−1.97	−4.39-0.45	0.108
Systolic BP (mmHg)	53	−3.26	−9.15-2.63	0.271	−3.38	−9.19-2.42	0.247	−4.00	−9.74-1.74	0.168	−4.09	−10.01-1.83	0.171
Diastolic BP (mmHg)	53	−1.43	−5.21-2.34	0.449	−1.94	−5.75-1.88	0.312	−2.13	−5.99-1.73	0.273	−2.17	−6.15-1.81	0.279
Insulin (mU/L)	44	1.22	−2.92-5.37	0.554	1.46	−2.52-5.44	0.462	1.34	−2.76-5.43	0.513	1.64	−2.54-5.82	0.432
Glucose (mmol/L)	45	−0.47	−1.11-0.17	0.143	−0.54	−1.18-0.10	0.095	−0.55	−1.20-0.11	0.101	−0.46	−1.08-0.17	0.149
HbA1c (%)	44	−0.29	−0.58-0.01	0.057	−0.20	−0.47-0.06	0.124	−0.22	−0.49-0.04	0.095	−0.23	−0.50-0.04	0.092
Cholesterol (mmol/L)	45	−0.22	−0.61-0.17	0.261	0.01	−0.37-0.38	0.969	0.03	−0.34-0.41	0.865	−0.03	−0.40-0.33	0.859
Triglycerides (mmol/L)	45	−0.26	−0.63-0.12	0.174	−0.22	−0.58-0.14	0.225	−0.22	−0.57-0.14	0.221	−0.26	−0.63-0.10	0.151
LDL (mmol/L)	44	−0.10	−0.45-0.24	0.544	0.08	−0.25-0.40	0.659	0.08	−0.25-0.41	0.635	0.07	−0.26-0.39	0.675
HDL (mmol/L)	45	−0.01	−0.11-0.08	0.760	0.00	−0.09-0.09	0.978	0.00	−0.10-0.09	0.946	−0.01	−0.11-0.08	0.801
CRP (mg/L)	44	−1.15	−4.72-2.41	0.517	−0.79	−4.36-2.77	0.655	−1.02	−4.52-2.49	0.560	−1.05	−4.66-2.56	0.559

Model 1 is crude and unadjusted. Model 2 is adjusted for age at baseline and the baseline assessment of the respective outcome measure. Model 3 is adjusted for the confounders in Model 2 plus kids in the household and employment status. Model 4 adjusts for all potential confounders in model 3 plus T2DM status.

Abbreviations: CI = confidence intervals; BMI = body mass index; Waist = waist circumference; Hip = hip circumference; HbA1C = glycated haemoglobin; LDL = low-density lipoprotein; HDL = high-density lipoprotein; CRP = C-reactive protein.

## Results

### Baseline characteristics

Tables 2 and 3 summarise participant characteristics at baseline, comparing the active and waitlisted groups. There were no statistical differences between the two groups at T1.

Tables 4 and 5 compare the baseline characteristics of participants by group and T3 follow-up status. There were no statistical differences in any of the baseline demographics or diabetes status between the participants lost to follow-up (LTF) and those who attended their T3 assessment from either group (Table 4). There were, however, statistical differences between the active participants LTF and those assessed at T3 in baseline age, cholesterol and CRP concentration. The women LTF from the active group were on average younger, with lower total cholesterol, triglyceride concentration, and LDL cholesterol and higher CRP concentrations (Table 5). Additionally, there was a statistical difference between those LTF and those assessed from the waitlisted group, those LTF on average weighed less, had a lower BMI, and smaller waist and hip circumferences (Table 5).

### Program fidelity and attendance

The 12-week program was implemented as intended for all three groups (Groups A, C and E). Key activities for each class were implemented as outlined in the facilitator’s manual. Class duration averaged 59 min. Session attendance by the active group was low. Participants were encouraged to attend two classes per week for twelve weeks. The average number of classes attended was 9.5 (95% CI, 7.4 - 11.6) of which 7 participants did not attend a single exercise class; 6 of these participants withdrew from the program and the other participant attended the T3 anthropometric assessment. Nutrition workshops also experienced low attendance. Of the four nutrition education workshops, the average number attended was 1 workshop, with 24 of the 51 active participants not attending a single nutrition workshop. The waitlisted participants also had low attendance at their workshops.

### Multivariable results

Regression analysis was conducted to determine the association between group (i.e. active or waitlisted) and the mean change in anthropometric and clinical assessments over time. Table 6 shows the difference from baseline to immediately post program (i.e. T1 - T2) and Table 7 shows the change between baseline and three months post program (i.e. T1 - T3). A negative coefficient indicated that the active group had a reduction in the outcome variable compared with the waitlisted group.

### Changes from baseline to immediate post program (T1-T2)

After adjusting for the baseline weight, age, children in the household, employment status and T2DM, there was a modest, yet statistically significant difference in weight within the active group compared to the waitlisted group from baseline to T2, ^-^1.65 kg (95% CI, ^-^3.27 - ^-^0.03) (Table 6). Similarly, there was a statistically significant difference in BMI of the active group compared to the waitlisted group from baseline to T2, ^-^0.66 kg/m^2^ (95% CI, ^-^1.27- ^-^0.05). These differences are similar across the models (see Table 6) indicating that the potential confounders had little effect on the results. The active group had decreases in waist and hip circumference, ^-^1.50 cm and ^-^0.84 cm respectively compared to the waitlisted group however this is within the 2% measurement error margin and was not statistically significant. The active group also had decreases in systolic and diastolic blood pressure relative to the waitlisted group although blood pressure was measured with low precision and these changes were not statistically significant however, it may be clinically significant (^-^1.24 mmHg and ^-^2.46 mmHg). CRP concentrations also changed from baseline to T2 for the active group relative to the waitlisted participants (^-^0.57 mg/L), however, this was not statistically significant. There is no difference between groups for fasting glucose, fasting insulin, HbA1c, total cholesterol, triglyceride concentrations, LDL cholesterol and HDL cholesterol.

### Changes from baseline to three months post program (T1-T3)

After adjusting for baseline weight, age, children in the household, employment status and T2DM the active group had a statistically significant reduction in weight compared to the waitlisted group from baseline to T3 (Table 7). The change in weight from baseline to T3 of the active group relative to the waitlisted group was ^-^2.50 kg (95% CI, ^-^4.46 - ^-^0.54). Similarly there was a statistically significant difference between baseline BMI and BMI at T3 between the active and waitlisted groups; ^-^1.03 kg/m^2^ (95% CI, ^-^1.79 - ^-^0.27). The relative difference in the change of waist and hip circumference between baseline and T3 of the two groups was not statistically significant but was ^-^1.50 cm and ^-^1.97 cm respectively. Systolic and diastolic blood pressure was also improved in the active group relative to the waitlisted group from baseline to T3 but did not reach statistical significance; ^-^4.09 mmHg (95% CI, ^-^10.01 - 1.83) and ^-^2.17 mmHg (95% CI, ^-^6.15 - 1.81) respectively. Other differences that were not statistically significant were fasting glucose, HbA1C, triglyceride concentration and CRP. There appeared to be no difference between groups for fasting insulin, cholesterol, LDL cholesterol and HDL cholesterol.

### Changes in weight

**Figure 2:**
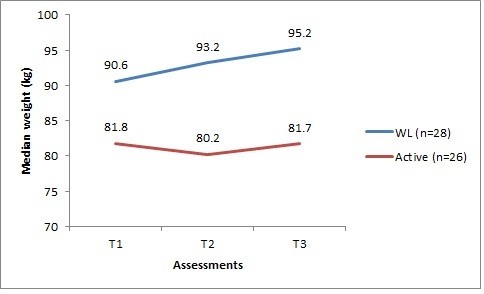
Median weight for the active and waitlisted participants with complete data from T1 to T3.

The median weight of the active and waitlisted groups was plotted at each assessment time point (Figure 2). Only participants weight measurements for all three time points were included. Participants lost to follow-up were on average heavier in the active group and lighter in the waitlisted group.

## Discussion

This study is a pragmatic randomised controlled trial of a 12-week physical activity and nutrition program with structured group exercise classes for Aboriginal and Torres Strait Islander women. Only limited research reports the results of interventions with a robust controlled design with measured endpoints, including follow-up 3 months after cessation of the intervention. Despite relatively low attendance rates for the program, we found a modest but significant weight loss in the active group compared to the control group, and favourable changes in blood pressure and other metabolic measures. The small number of participants in this trial might well explain the lack of statistically significant results in some of these outcome measures.

Research consistently shows a strong association between weight loss and reduction in blood pressure [6,7]. A meta-analysis of 25 trials found for each kilo of weight lost through energy restriction, increased physical activity or both, systolic blood pressure reduced by 1.05 mmHg (95% CI, 1.43 - 0.66) and diastolic blood pressure by 0.92 mmHg (95% CI, 1.28 - 0.55) [7].

Exercise interventions for weight loss have had mixed results. Most studies have reported modest weight loss (1–3 kg) even though the studies tended to be more intensive, with higher compliance and longer trial periods than this study [5,19]. A meta-analysis of 43 randomised control trials using exercise as the primary means for weight loss found that exercise combined with diet resulted in a weight loss of 1.1 kg, and that increasing the intensity of the exercise program increased weight loss (1.5 kg). Interventions including only an exercise component resulted in less weight loss [5]. Our study is the first to report the results of a group intervention study including twelve weeks of structured physical activity and nutrition education sessions for Aboriginal and Torres Strait Islander women living in an urban setting.

A 10-week community exercise and nutrition program (CHAPP) targeting obese African American adults found a smaller effect on body mass at the three month follow-up, with a loss of 1.3 kg. The effect on diastolic blood pressure was similar, ^-^2.8 mmHg, but they achieved a greater change in systolic pressure ^-^5.8 mmHg [13]. Compared to a 4-month program targeting obese Maori men and women in New Zealand our weight loss was less, 2.5 kg compared to 3.1 kg (95% CI, ^-^4.0 - ^-^2.1) [12]. The study of Maori men and women also found a larger improvement in systolic blood pressure, reduced by 7 mmHg (95% CI, 13–1) but less of an improvement in diastolic blood pressure, reduced by 1 mmHg (95% CI, ^-^4 - ^-^2) [12]. The 8-week pilot study, the Thursday Island Women’s Fitness Challenge, did not find any weight loss and blood pressure was not assessed. However they did find a reduction in waist circumference, of 4.93 cm, which was not replicated in our trial [14]. These studies had a small number of participants (n = 70, 30, and 10 respectively) and are pre-post designs with no control group. Thus, there is the potential for selection bias in these study findings [12].

This study has limitations, most significantly the large amount of missing data. The project implemented many actions to improve data collection. Different modes of collection were offered, including home or office visits, to conduct anthropometric assessments and collect fasting blood samples. However, appointments were still difficult to organise and were often cancelled or rescheduled. Only one-half of participants attended their T3 anthropometric assessment and slightly less completed their pathology test. This significantly reduced the sample size.

Program ‘dose’ was another issue. Attendance at the exercise classes and nutrition workshops was low. Participants in the active group attended on average 40% of the recommended group exercise sessions. This situation is likely to have reduced the effectiveness of the program. Thus, the findings presented here are a conservative picture of the potential effectiveness of this intervention. To gain insight into the factors influencing participation, a qualitative analysis of post-program interviews is being undertaken and a paper will be forthcoming.

The project also experienced difficulties in the assessment of waist circumference and blood pressure. The ISAK accredited assessors’ accuracy with assessing waist circumference in this difficult-to-measure group is unknown. The assessors were conscious to be sensitive and appropriate with the participants and in some cases it was not appropriate to palpate for landmarks. The assessors also had some difficulties ensuring the tape measure was exactly horizontal. Additionally, some participants chose not to remove items of clothes from around their waist and hips, consequently their assessments were performed over their clothing. The large arm cuff for the Ormon blood pressure machine was ill-fitting on upper arms of different shapes and consequently gave some error readings which resulted in some missing data for some of the participants with high BMIs.

## Conclusions

The Aboriginal and Torres Strait Islander Women’s Fitness Program found modest reductions in weight, BMI and blood pressure, which were evident at the immediate post program assessment with further improvement at the three month follow-up assessment. Changes in the primary outcome measure, waist circumference, and metabolic measures were not evident. Longer term follow-up of all participants would be valuable, as similar programs undertaken in other populations show mixed results, especially over the longer term.

## Competing interests

The authors declare that they have no competing interests.

## Authors’ contributions

KC developed and implemented the pilot study. KC, RM, MC and AE were all responsible for developing the research project. KC carried out the analysis under the guidance of AE, ML and KD, who also assisted in the interpretation of the results. KC produced the first draft. All authors were responsible for the critical review of the article. All authors read and approved the final manuscript.

## Additional information

The project protocol is published in BMC Public Health and can be accessed at http://www.biomedcentral.com/content/pdf/1471-2458-11-655.pdf.

## Pre-publication history

The pre-publication history for this paper can be accessed here:

http://www.biomedcentral.com/1471-2458/12/933/prepub
